# Flexible growing rods: a biomechanical pilot study of polymer rod constructs in the stability of skeletally immature spines

**DOI:** 10.1186/s13013-016-0087-6

**Published:** 2016-09-23

**Authors:** Donita I. Bylski-Austrow, David L. Glos, Anne C. Bonifas, Max F. Carvalho, Matthew C. Coombs, Peter F. Sturm

**Affiliations:** 1 Orthopaedics, Cincinnati Children’s Hospital Medical Center, Cincinnati, OH 45229-3039 USA; 2 University of Cincinnati, Cincinnati, OH USA

**Keywords:** Early onset scoliosis, Growing rods, Spine instrumentation, Biomechanics, Range of motion, PEEK rods, Polymer, Polyetheretherketone, Titanium, Cobalt chrome alloy

## Abstract

**Background:**

Surgical treatments for early onset scoliosis (EOS) correct curvatures and improve respiratory function but involve many complications. A distractible, or ‘growing rod,’ implant construct that is more flexible than current metal rod systems may sufficiently correct curves in small children and reduce complications due to biomechanical factors. The purpose of this pilot study was to determine ranges of motion (ROM) after implantation of simulated growing rod constructs with a range of clinically relevant structural properties. The hypothesis was that ROM of spines instrumented with polymer rods would be greater than conventional metal rods and lower than non-instrumented controls.

**Methods:**

Biomechanical tests were conducted on six thoracic spines from skeletally immature domestic swines (35–40 kg). Paired pedicle screws were used as anchors at proximal and distal levels. Specimens were tested under the following conditions: control, then dual rods of polyetheretherketone (PEEK) (diameter 6.25 mm), titanium (4 mm), and cobalt-chrome alloy (CoCr) (5 mm). Lateral bending (LB) and flexion-extension (FE) moments were applied, and vertebral rotations were measured. Differences were determined by two-tailed t-tests and Bonferroni for four primary comparisons: PEEK vs control and PEEK vs CoCr, in LB and FE (α = 0.05/4).

**Results:**

In LB, ROM of spine segments after instrumenting with PEEK rods was lower than the non-instrumented control condition at each instrumented level. ROM was greater with PEEK rods than with Ti and CoCr rods at every instrumented level. Combining treated levels, in LB, ROM for PEEK rods was 35 % of control (*p* < 0.0001) and 270 % of CoCr rods (*p* < 0.01). In FE, ROM with PEEK was 27 % of control (*p* < 0.001) and 180 % of CoCr (*p* < 0.01). At proximal and distal adjacent non-instrumented levels in FE, mean ROM was lower for PEEK than for either metal.

**Conclusions:**

PEEK rods increased flexibility versus metal rods, and decreased flexibility versus non-instrumented controls, both over the entire instrumented segment and at each individual level. Smaller mean increases in ROM at proximal and distal adjacent motion segments occurred with PEEK compared to metal rods, which may help decrease complications, such as junctional kyphosis. Flexible growing rods may eventually help improve treatment options for young patients with severe deformity.

## Background

**Fig. 1:**
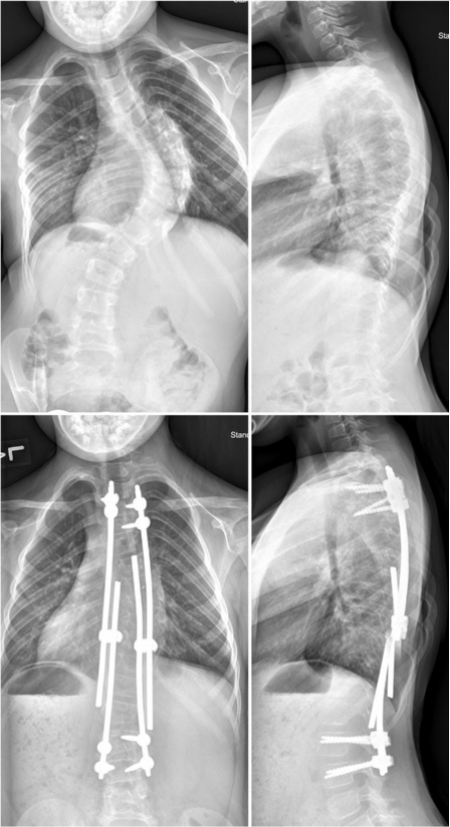
Radiographs of a patient with early onset scoliosis, preoperative (*top*) and after implantation of growing rod construct (*bottom*). (*Left*) Posterior-anterior view. (*Right*) Sagittal view

Early onset scoliosis (EOS) presents before the age of 10 years [1] and is associated with high morbidity and mortality rates compared to adolescent idiopathic scoliosis (AIS) due to chest wall deformities that restrict pulmonary development [2]. Fusion of thoracic spinal deformities at this early age is contraindicated [3, 4]. Current treatments include serial casting [5–7], but conservative methods are not always effective. Surgical treatments include spine distraction and rib expansion [2]. Distractible ‘growing rods’ (Fig. 1) have been used for several decades in the attempt to control both the spinal deformity and to allow for spinal growth [8] and have reported to be effective [9–14]. Treatment goals in EOS include minimizing spinal deformity over the life of the patient, the extent of any final spinal fusion, complications, procedures, hospitalizations, and burden for the family; and maximizing thoracic function including motion of the chest and spine [1].

Surgical treatments typically require multiple surgeries and involve many complications, including infection, instrumentation failures, corrosion, joint fusion, and changes to adjacent motion segments [13, 15]. Complications of growing rod treatment for EOS were reported [15]. In a study of 141 patients, the investigators concluded that management of EOS is prolonged regardless of treatment modality, and so complications are frequent and expected. Complications may be reduced by delaying initial implantation when possible, using dual rods, and limiting the number of lengthening procedures. Early changes of the thoracic geometry after implantation of a growing rod were shown to have a corrective effect on chest wall geometry [16]. Constructs that lengthen magnetically reduce the number of surgeries [17], but the instrumentation is stiff, the elongating section cannot be contoured, and MRI is contraindicated [18]. Construct mechanical properties, therefore, affect both treatment efficacy and some of the complications.

Early biomechanical studies have been reported [19]. In one study, distraction of long non-segmental spinal constructs was shown to result in load-sharing across multiple levels, rather than a local concentration of distractive effects, during a simulated distraction maneuver [20]. A foundation composed of four pedicle screws implanted in two adjacent vertebral bodies provided a stronger construct in pullout tests compared to laminar hooks [21], and cross-links were not shown to enhance fixation. Spine versus rib anchors have also been assessed in biomechanical tests [22]. A pediatric cadaveric study [23] reported differences in distraction failure forces due to anchor points on ribs, laminae, or pedicles. The effect of distraction force [24] and timing [25] were explored using computer models. Development of a “smart” growing rod system has been proposed [26].

The immature porcine spine has been reported to provide a reasonably similar growth rate and anatomical dimensions to the EOS patient population [19]. An in vivo study in swine showed an increase in vertebral body height in distracted segments compared to non-distracted control segments [27]. In a clinical case series, growing rod treatment performed with lengthening procedures every 6 months was reported to stimulate growth in vertebrae within the instrumented levels [28]. Length gains, however, tend to decrease with time. These “diminishing returns” have been attributed to auto-fusion of the spine from prolonged immobilization by a rigid device [29]. The force required to distract the spine doubled by the fifth lengthening in a study on EOS patients in which distraction forces were measured during lengthening procedures [30].

Some of the complications stem from mechanical factors. Rod fractures, a relatively common complication, may be related to the significant increase in distraction force over time. In a retrospective review of a multicenter database, rod fracture occurred in 15 % of patients [31]. The high stiffness of conventional metal rods creates compliance mismatches between spine and instrumentation, stress concentrations, and motion redistribution, factors which likely contribute to rod breakage, screw pull-out, auto-fusion, and junctional kyphosis [15, 32, 33]. Using a computational model, a more flexible non-fusion correction system for AIS which used non-locking polyaxial pedicle screws and mobile connectors was reported to reduce intervertebral rotation less than more rigid implants [34]. Growing rods with a telescopic sleeve component have been designed to reduce constraints to axial rotation, with the expectation that growth would be allowed while maintaining the axial flexibility of the spine for improved capacity for final correction [35].

Growing rods with greater flexibility might result in a sufficiently straight and more flexible spine with fewer surgical complications. The polymer polyetheretherketone (PEEK) has a lower modulus than traditional rod materials, which might allow for greater range of motion (ROM) than standard metal cobalt-chrome alloy (CoCr) or titanium (Ti) rods. The bending stiffness of PEEK is about 10 % of a titanium rod of the same diameter [36]. Rods made of PEEK have been previously reported for use in adult, short segment, lumbar spine surgery. In cadaveric tests, short PEEK rods provided comparable stability to titanium rods of equivalent diameter [37, 38]. PEEK rods have been shown to affect disc pressure in levels adjacent to spinal instrumentation [39]. To the investigators’ knowledge, no prior biomechanical study was performed on PEEK rods of the length of the thoracic spine. A preliminary report suggested that PEEK rods of dimensions suitable for EOS patients might provide sufficient stability to correct a curve and withstand physiological loads, at least in very small children [40]. No previous report has presented effects of rods of different material properties on the motion of each intervertebral joint, in particular, motion at the adjacent non-instrumented segments.

Therefore, the purpose of this study was to determine changes to the biomechanical properties of skeletally immature spines after implantation of simulated growing rod constructs with a range of clinically relevant structural properties. The primary hypotheses were that ROM of spines instrumented with PEEK rods are 1) lower than non-instrumented controls, 2) greater than metal rods, and 3) closer to controls than to metal rod constructs. Further, adjacent segment motion was expected to be lower with polymer rods compared to conventional systems.

## Methods

In vitro biomechanical tests were conducted on six porcine thoracic spines harvested from skeletally immature Yorkshire cross pigs (10–14 weeks of age weeks of age, body mass 35–40 kg). The spines were obtained after death from animals that had been previously utilized for other studies that had not involved the spine (approved by IACUC, University of Cincinnati). Spines were sectioned to include vertebrae T1-T13 (domestic pigs have 14 to 15 thoracic vertebrae), then were frozen at -20 °C until testing. To prepare test specimens, muscle was carefully removed to preserve ligaments, joint structures, transverse processes, and rib articulations. Paired pedicle screws (polyaxial, 5.0 X 35 mm, Ti; DePuy Spine, Raynham MA) were inserted into T3 and T4 for the proximal anchors, and into T10 and T11 for the distal anchors. A non-instrumented intervertebral joint remained above and below the upper and lower instrumented vertebrae. Pedicle screws were inserted freehand. The entry point was prepared using an awl at the junction of a line between the transverse process and lateral border of the pars. The pedicle canal was created using a pedicle probe. The pedicle wall integrity was verified using a ball-tip probe before inserting each screw.

The specimens were carefully aligned in neutral orientation while potting the specimen into end blocks in fiberglass-reinforced resin (Bondo, St. Paul, MN) to facilitate reproducible positioning into the loading device. For flexion-extension testing, specimens were placed in the system with the caudal and cranial end-blocks level with the base. Testing was performed at room temperature and specimens were kept moist using physiological saline solution.

**Fig. 2:**
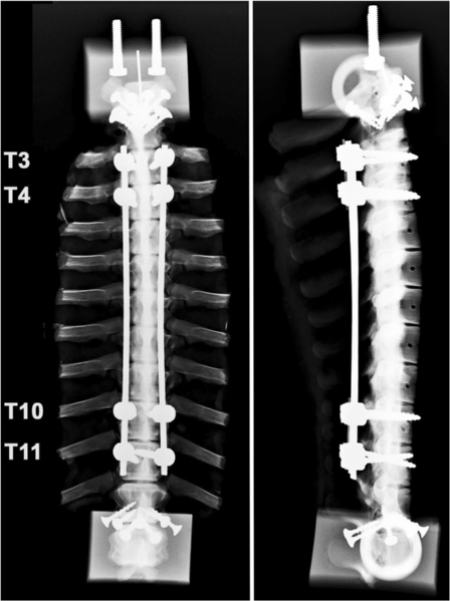
Spine test specimen with dual titanium rod construct. *Left*: Coronal view (Reproduced from Reference 40, Fig. 1: Bylski-Austrow DI, Glos DL, Bonifas AC, Carvalho MF, Coombs MT, Sturm PF. Flexible growing rods: A pilot study to determine if polymer rod constructs may provide stability to skeletally immature spines. Scoliosis 2015, 10(Suppl 1):O73.) *Right*: Sagittal view. The rods were anchored using two pairs of pedicle screws at the proximal end, and a second set of two pairs at the distal end. The discs adjacent to the instrumented region at each end were not instrumented

**Fig. 3:**
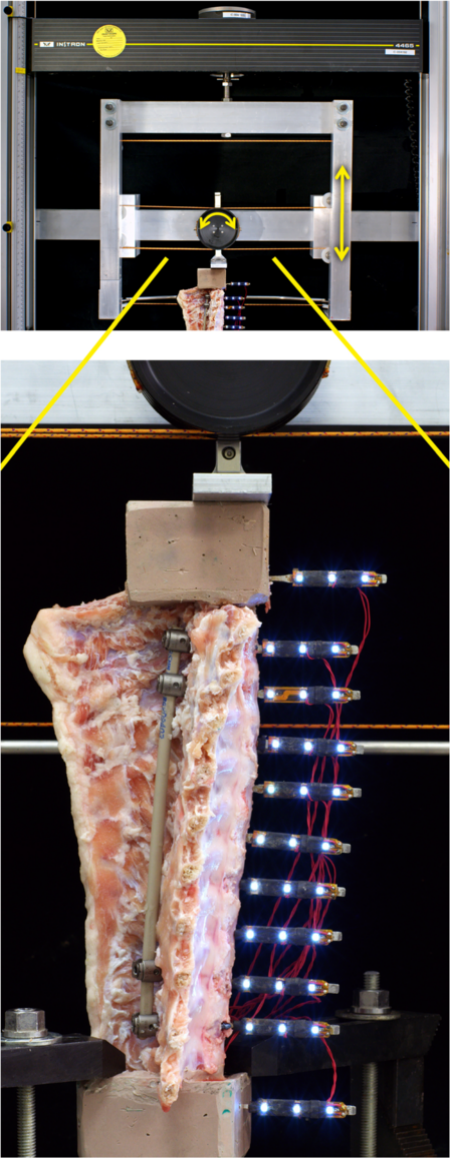
Spine test specimen with PEEK rod construct mounted for a flexion-extension test. *Bottom*: At each vertebra, a marker array with 3 white LEDs was inserted into the anterior aspect for video motion analysis (Reproduced from Reference 40: Bylski-Austrow DI, Glos DL, Bonifas AC, Carvalho MF, Coombs MT, Sturm PF. Flexible growing rods: A pilot study to determine if polymer rod constructs may provide stability to skeletally immature spines. Scoliosis 2015, 10(Suppl 1):O73.) *Top*: A floating pulley system was used to convert linear displacement of crosshead to rotation to apply moment to the cranial end of the specimen

Each specimen was tested before and after instrumentation using a repeated measures experimental design. The order of testing was: 1) before rod insertion (Control), followed by 2) PEEK rods (6.25 mm diameter, *n* = 6, Quadrant Plastics, Fort Wayne IN) (Fig. 2), 3) titanium rods (*n* = 6, 4 mm diameter, Synthes, Paoli PA), and 4) cobalt-chrome-molybdenum alloy rods (CoCr) (*n* = 4, 5.5 mm diameter, DePuy Spine, Raynham MA) (Fig. 2, radiograph with CoCr rods). The rods were approximately 200 mm long, and straight in both coronal and sagittal planes for this pilot study, as PEEK cannot be contoured at room temperature.

Tests were conducted in lateral bending (LB) followed by flexion-extension (FE). Moments of ±5 Nm were applied using a materials test system (Instron 4465; Instron, Norwood, MA) with control and data acquisition software (TestWorks 4; MTS, Eden Prairie, MN) and a custom cable-floating pulley fixture (Fig. 3). The system allowed for continuous cycling from full flexion to full extension, or left to right lateral bending, as previously described [32]. Specimen motion was largely in the plane. However, coupled motions were allowed and the rotational axes were not prescribed. Loads were measured using the load cell (5 kN) of the test system. Displacements of vertebrae and mounting blocks were recorded using high definition video (Nikon D7000, with Tokina At-X Pro Macro 100 F2.8 D lens; Nikon, Tokyo, Japan). Five cycles were applied at a frequency of 0.10 Hz for FE or 0.05 Hz for LB, using a sinusoidal waveform. The fourth cycle was analyzed.

Vertebral orientation at each level was determined from a triplet LED array which was rigidly pinned to each vertebra. Sampling frequency was 24 Hz, as was the video frame rate. Tests were performed with room lights off to allow for ease of distinguishing markers from background. Rotations were calculated using a customized program (Mathworks, MATLAB R2011b, MathWorks, Natick, MA) [41]. Range of motion, determined from the moment-rotation curve for each motion segment, was defined as the maximum side-to-side rotation. Range of motion over the entire treated region was determined by adding the ROM at each instrumented level (T3-T4 to T10-T11).

Statistical differences between treatments in ROM over the instrumented segments were determined by two-tailed paired t-tests and Bonferroni correction. Four primary comparisons were used: PEEK vs control and PEEK vs CoCr, in LB and FE (α = 0.05/4 = 0.0125).

## Results

**Fig. 4:**
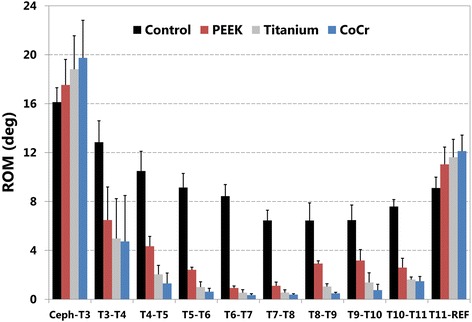
Range of motion (ROM) in lateral bending at each motion segment for each rod type. Control non-instrumented condition, and with dual rods of polyetheretherketone (PEEK), Titanium, and Cobalt-Chrome alloy (CoCr) are shown. The instrumented levels spanned T3-T4 through T10-T11. Ceph-T3 indicates the proximal adjacent level, and T11-REF the distal adjacent level

**Fig. 5:**
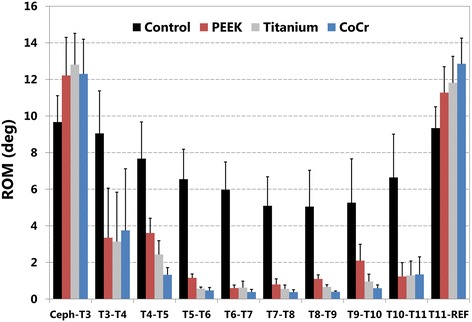
Range of motion (ROM) in flexion-extension at each motion segment for each rod type. Control non-instrumented condition, and with dual rods of polyetheretherketone (PEEK), Titanium, and Cobalt-Chrome alloy (CoCr) are shown. The instrumented levels spanned T3-T4 through T10-T11. Ceph-T3 indicates the proximal adjacent level, and T11-REF the distal adjacent level

For non-instrumented control spines, ROM in LB (Fig. 4) and in FE (Fig. 5) gradually decreased from proximal to mid-thoracic segments, then increased from mid- to lower thoracic levels. Control values for mean ROM in LB ranged from 6° at T7-T8 to 16° at T2-T3, the proximal adjacent segment. In FE, ROM ranged from 5° at mid-thoracic to 10° at the proximal adjacent level. For all three instrumented conditions, the smallest ROM values, less than 1° in both LB and FE, were at mid-construct, and all three showed large differences in ROM across the proximal and distal junctions, 8° to 15°, compared to the differences of 1° to 3° in the control condition.

In lateral bending, ROM after each treatment, including PEEK rods, was lower than non-instrumented control at every instrumented level (Fig. 4). Range of motion was greater with PEEK rods than for Ti or CoCr rods at every instrumented level. Conversely, at the proximal and distal non-instrumented segments of the instrumented specimens, ROM was greater for every instrumented condition compared to the control condition, and the order was reversed. That is, at both proximal and distal non-instrumented levels, mean ROM was lowest for control, then PEEK, Ti, and CoCr. The largest difference in ROM between adjacent levels, 15°, was between the upper instrumented vertebra and first proximal adjacent level with Co-Cr rods.

In flexion-extension, ROM after each treatment, including PEEK rods, was lower than non-instrumented control at every instrumented level (Fig. 5). Range of motion was usually greater with PEEK rods than Ti or CoCr rods at individual levels, but variability was greater in FE than in LB. Mean ROM at proximal and distal non-instrumented levels was at least slightly lower for PEEK than for Ti and CoCr. At the distal adjacent segment, but not the proximal adjacent, the pattern of mean ROM was reversed compared to instrumented levels, as with LB. The largest difference in ROM between adjacent levels, 11.5°, was between the lowest instrumented vertebra and first distal adjacent level with Co-Cr rods.

**Fig. 6:**
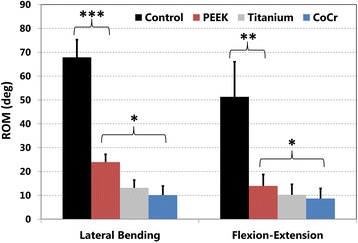
Range of motion (ROM) of entire instrumented region in lateral bending (left) and flexion-extension (right). Control non-instrumented condition, and with dual rods of polyetheretherketone (PEEK), Titanium, and Cobalt-Chrome alloy (CoCr) are shown. *** *p* < 0.000125, ** *p* < 0.00125, * *p* < 0.0125 (α = 0.05/4 = 0.0125). (Reproduced from Reference 40, Bylski-Austrow DI, Glos DL, Bonifas AC, Carvalho MF, Coombs MT, Sturm PF. Flexible growing rods: A pilot study to determine if polymer rod constructs may provide stability to skeletally immature spines. Scoliosis 2015, 10(Suppl 1):O73.)

The ROM over all of the instrumented segments in lateral bending (Fig. 6) for each condition were: Control 67.9° (±7.4°), PEEK 23.9° (±3.3°), Ti 13.1° (±3.3°), CoCr 10.1° (±3.8°). Differences between Control and PEEK (*p* < 0.0001) and PEEK and CoCr (*p* < 0.002) were both significant. Over the instrumented levels, ROM for spines with PEEK rods was 35 % of non-instrumented controls, and 2.7 times greater than spines with CoCr rods. For flexion-extension (Fig. 6), the total ROM of each motion segment within the instrumented segment for each test group was: Control 51.3° (±14.7°), PEEK 13.9° (±4.8°), Ti 10.2° (±4.4°), CoCr 8.6° (±4.3°). Differences between control and PEEK (*p* < 0.0005) and PEEK and CoCr (*p* < 0.005) were both significant. Over the instrumented levels, ROM for spines with PEEK rods was 27 % of non-instrumented control, and 1.8 times greater than spines with CoCr rods.

At the proximal and distal adjacent discs in LB and FE, ROM was always greater for CoCr than for PEEK. The mean difference in ROM between PEEK and CoCr was 0.9° (± 0.5°). Peak-to-peak moment (∆M) for each group, Control, PEEK, Ti, and CoCr, respectively, were, in LB: 10.7 Nm (± 0.28 Nm), 10.7 Nm (± 0.26 Nm), 10.9 Nm (± 0.56 Nm), 10.7 Nm (±0.37); and in FE: 10.8 Nm (0.39 ± Nm), 10.9 Nm (± 0.40 Nm), 10.8 Nm (± 0.32 Nm), 10.6 Nm (±0.25). No differences were found in applied moments between groups (*p* > 0.25), and the target maximum moment was met in all cases of each condition in both loading directions.

## Discussion

The structural properties of the rods were shown to significantly affect the biomechanical properties of the spine in a simulated growing rod construct. Range of motion of spines instrumented with PEEK rods was closer to that of metal rods than to that of the control, non-instrumented condition. Range of motion with PEEK rods was 27 to 35 % of control. By contrast, ROM with PEEK rods was 1.8 to 2.7 times greater than with Co-Cr rods. Therefore, results supported the first two hypotheses, as the mean ROM with PEEK rods was between the control condition and the metal rods. The third hypothesis, which was based on the very high flexibility of single, isolated, PEEK rods, was not supported. The polymeric growing rod constructs when implanted as dual rods did, in fact, very significantly decrease thoracic spine motion compared to the control condition. Further, smaller increases in mean ROM of adjacent discs compared to control usually, but not always, occurred with PEEK compared to the metal rods, specifically at the distal end in FE and at both proximal and distal ends in LB.

The magnitude of the ROM at adjacent discs, and the differences between the ROM between the first instrumented motion segment and the adjacent disc, may be expected to affect the risk of junctional kyphosis [4]. However, all rod types, when anchored with two pairs of pedicle screws at each end, created relatively large changes in motion across the junctions. Range of motion of the adjacent disc was always greater for CoCr than for PEEK. Whether a magnitude of difference of 1° is clinically significant, however, is not yet known.

Limitations of this study include *in vitro* tests on physiologically normal quadruped spines. The use of porcine spines, without ribs, for *in vitro* studies using pedicle screws and transverse process hook anchors has been reviewed [19, 32]. The lack of a rib cage certainly decreased the stiffness of the thoracic spine. For the specific aim of this study, to determine if polymer rods might provide increased stiffness to the spine in a construct, the use of the isolated spine was simpler and conservative. That is, because the PEEK rods clearly provided increased support to a thoracic spine without the rib cage, it would also do so for the stiffer structure of a spine plus the intact rib cage. However, no model can mimic very well the severe deformities of the spine and thorax of a young child with EOS.

Other limitations include that specimen numbers were relatively small, especially for the Co-Cr condition. Physiological loads of body weight, activity, and curve correction in the relevant patient populations are not yet well defined. The two rods were each intact, and did not contain any distraction mechanism. Further tests are needed in buckling and torsion, and strength and fatigue properties are essential. Total ROM was defined as the sum of individual motion segment ROMs, which is not necessarily the same ROM if it were determined by directly measuring the ROM within the instrumented levels, due to differences in timing of motion along the spine caused by loading method and specimen viscoelasticity. The boundary or constraint conditions used, as well as the loading method, may affect ROM patterns to some extent. The inability to plastically deform PEEK to contour the initial rod configuration in situ to better approximate a desirable sagittal profile is a limitation of this material. Magnetically controlled growing rods that are remotely lengthened using an actuator without additional surgeries are a recent advance in the field. Other implant structural factors that may be considered are composite structures that include a partial PEEK rod as part of a magnetically controlled construct, tapered rod diameter, and novel connector designs.

Specific results will depend on material and geometric properties. Rod length was the same for all conditions, but material and diameter both changed. This set of conditions was chosen to reflect a range of structural properties. The extremely low bending stiffness of single isolated PEEK rods suggested that not even these relatively large diameter rods would form a construct much less flexible than the intact isolated thoracic spine. In a study of comparative mechanical properties of commonly used spinal rods [36], the stiffness of PEEK rods was only 4 % that of Ti rods of the same diameter, whereas carbon fiber reinforced PEEK was close to titanium. That study also reported that the effect of mechanical property differences increased with decreasing rod diameter. Therefore, the present investigators did not expect *a priori* that even a relatively large diameter PEEK rod would substantially decrease ROM compared to control. The larger diameter PEEK rod, clinically relevant Co-Cr rod, and the small diameter titanium rod used in the present study provided a range of properties, whereas the primary comparisons were between the Co-Cr and PEEK. Differences among the moduli of the materials was the primary factor affecting bending stiffness differences, whereas the diameter was a secondary factor, over a range of test conditions which spanned, and slightly exceeded, the physiologically relevant range.

Potential advantages of PEEK for implants include high biocompatibility, fatigue resistance, and lower modulus than titanium. Lower stiffness imparts greater load sharing with the anterior column, reduced stress at the bone-to-screw interface, and reduced beam scattering artifact in MRI and CT [37, 42–45]. Titanium induces significant artifacts on CT or MRI which constrain post-operative assessment of adjacent structures, whereas PEEK, without addition of compounding material, is radiolucent, neither distorting nor visible in MRIs [46]. A biomechanical study of a lumbar fusion construct concluded that segments instrumented with PEEK rods more closely mimicked intact physiologic loading in the subadjacent level than titanium [47]. PEEK was reported to be relatively inert biologically with no evidence of inflammatory reaction to wear debris [48].

Possible adverse effects of PEEK rods in temporary, long-rod, non-fusion constructs may include lower deformity correction, loss of initial correction, higher infection rate, and elastic, high deformation, failure mode. Potential disadvantages of PEEK rods might be discerned, in part, from those reported for different but related uses. Clinical outcome studies on commercially available flexible fusion-promoting systems have shown higher failure rates with early reoperations compared to traditional metal fusion-promoting constructs [37, 49, 50]. In a retrieval analysis of explanted PEEK rods used for lumbar fusion in which 11 of 12 PEEK rod systems were employed for fusion at one level, and motion preservation at the adjacent level, no cases of PEEK rod fracture or pedicle screw fracture were noted. Permanent indentations by the set screws and pedicle screws were the most prevalent observations on the surface of explanted PEEK rods [51, 52]. Further studies quantifying wear debris and biological effects in a typical application are required. Polyetheretherketone may have increased bacterial activity on its surface compared to titanium [51, 53, 54]. In fatigue tests, PEEK was shown to be notch-sensitive [55]. Cyclic deformation was predominately elastic in the lifetime range [56]. Those authors concluded that the clinical significance was the potential for gross failure of PEEK implant devices without any substantial period of detectable difference in structural behavior. Therefore, for any application of PEEK to growing rods, design-related stress concentrations would require careful consideration.

The results of this pilot study did not rule out PEEK as a possible rod material for growing rods or, perhaps more likely, as a component of a composite structure. To the investigators’ knowledge, this is the first study to test polymers for applications in EOS. Plans for the next phase of the study include a set of components that comprise a full, clinically relevant, construct.

## Conclusions

In a biomechanical pilot study, simulated growing rod constructs using polymer rods provided greater stability compared to controls, greater flexibility compared with cobalt-chrome, and a more gradual motion and stiffness transition across junctions than conventional rods. This is a first feasibility study. A number of other design changes are possible and many additional preclinical tests would be necessary prior to translation of this concept. However, results showed that polymers may become a part of better treatment options for EOS. Maintenance and retention of greater spine flexibility would likely allow for fewer complications and higher satisfaction for patients, parents, and caregivers.

## Abbreviations

∆: Delta, difference
AIS: Adolescent idiopathic scoliosis
Co-Cr: Cobalt-chrome-molybdenum alloy
EOS: Early onset scoliosis
FE: Flexion-extension
LB: Lateral bending
LED: Light emitting diode
Nm: Newton-meters
PEEK: Polyetheretherketone
ROM: Range of motion
T1–T13: Thoracic vertebral levels
Ti: Titanium
